# Variants in glycine decarboxylase activate catabolic mechanisms of mitochondrial energy metabolism in the brain

**DOI:** 10.1016/j.jbc.2026.113098

**Published:** 2026-04-27

**Authors:** Alejandro Lopez-Ramirez, Andrew J. Worth, Ziyi (Lindsay) Wang, Saliha Yilmaz, Sebastian Hayes, Hsin-Yao Tang, Joseph Farris, Md Suhail Alam, Prasad Padmanabhan, Kasturi Haldar

**Affiliations:** 1 Boler-Parseghian Center for Rare and Neglected Diseases, Notre Dame, Indiana, USA; 2 Department Biological Sciences, University of Notre Dame, Notre Dame, Indiana, USA; 3 Agios Pharmaceuticals, Cambridge, Massachusetts, USA; 4 The Wistar Institute, Philadelphia, Pennsylvania, USA; 5 Center for Individualized Medicine, Mayo Clinic, Rochester Minnesota, USA

**Keywords:** astrocytes, brain, fatty acid oxidation, GLDC, metabolism, non-ketotic hyperglycinemia

## Abstract

Brain energy metabolism is produced from glucose by mitochondrial oxidative phosphorylation. Variants in the mitochondrial enzyme glycine decarboxylase (GLDC) cause a rare neurological disease, nonketotic hyperglycinemia, with expected hallmarks of brain glycine elevation and responsiveness to folate deficiency but the consequences for energy mechanisms remain unknown. We find that brains of young-attenuated mutant mice show a 1.5-fold increase in glycine and no change in folate responsiveness. They are, however, reduced > 5-fold in GLDC, indicate decrease in the mitochondrial lipoyl-transfer protein GCSH and lipoylation of the pyruvate dehydrogenase complex as well as rise in signatures of astrocyte mitochondrial β-oxidation of fatty acids proportionate to mutation severity and activation of pyruvate dehydrogenase. Together these data reveal a novel GLDC mechanism that regulates catabolic mitochondrial energy processes in both attenuated and severe brain disease and suggest new targets in energy metabolism to treat nonketotic hyperglycinemia.

The brain requires high levels of energy, utilizing a fifth of the total required by the body (1). Approximately 95% of the brain’s energy comes from glucose (1, 2, 3, 4). Oxidative phosphorylation of glucose *via* mitochondria produces acetyl co-enzyme A (acetyl-CoA), the key precursor for energy. Production of acetyl-CoA from pyruvate is catalyzed by dephosphorylation and activation of mitochondrial pyruvate dehydrogenase (PDH). However, the brain glucose levels provide neurons with only a few minutes of energy. Astrocytes, which are primary glial cells, also provide energy to neurons (5, 6, 7, 8). Lactate from astrocytes is an important energy source delivered to neurons *via* the astrocyte-neuron lactate shuttle (ANLS) (2, 9, 10). Unlike neurons, astrocytes have the capacity to activate mitochondrial β-oxidation of fatty acids as an alternate energy supply (11, 12, 13, 14, 15, 16). Neurodegenerative disease weakens both glucose metabolism and mitochondrial oxidative phosphorylation in the brain (17). In response, astrocytes, rather than dephosphorylating and activating their PDH complex, instead increase levels of glycolysis to build up lactate (18), which is then transferred *via* the ANLS, to boost neuronal levels of lactate and pyruvate that combined with dephosphorylation and activation of the neuronal PDH complex, results in increased ATP production *via* oxidative phosphorylation (18).

Nonketotic hyperglycinemia (NKH) is a rare neurometabolic disorder caused by a defect in the glycine cleavage system (GCS), which is a major pathway for glycine catabolism. The global prevalence of NKH is estimated at one in 76,000 (19). Approximately 80% of NKH is caused by a defect in glycine decarboxylase (GLDC). NKH is a complex, poorly understood disease with a wide range of clinical severity and over 50 different symptoms (20, 21). Patients with attenuated mutations and disease are affected by developmental delay, poor feeding, hypotonia, seizures, and infrequent death (22, 23). In contrast, those with severe disease display intractable seizures, loss of consciousness, severe hypotonia and frequent death. All of these symptoms have been ascribed to elevation of glycine (19, 21) (https://www.ncbi.nlm.nih.gov/books/NBK556140/, https://www.ncbi.nlm.nih.gov/books/NBK1357/). However, in murine models, elevation of brain glycine alone is insufficient to induce neurological disease or hypotonia (24, 25), suggesting additional mechanisms under lie attenuated and severe neurological disease. Deficiency in GLDC also disrupts the formation of 5, 10-methyl tetrahydrofolate (5,10-meTHF), a key intermediate in folate metabolism that gives rise to fatal, prenatal hydrocephalus (26). It is well-accepted that folate deficiency is associated with severe mutations (in humans and mice), but its involvement in attenuated mutations is poorly understood.

Neurological disease with symptoms of hypotonia and seizures are frequently associated with mitochondrial disorders with defects in enzymes of energy metabolism (27). However, whether GLDC mutational defects impact mitochondrial energy mechanisms in the brain is not known. GLDC has over 200 disease variants (mutations). In prior studies, we developed large scale genotype–phenotype studies of human variants and quantitative tools to comprehensively predict and separate effects of severe *versus* attenuated mutations (20). We successfully validated these predictions using CRISPR Cas9 to engineer mice with attenuated (*Gldc*^ATT/ATT^) and severe highly pathogenic mutations (*Gldc*^SEV/SEV^) and thereby develop humanized models of NKH disease (28) that we used in this study to investigate new mechanisms of metabolic changes in the NKH brain.

GLDC is present in all major regions of the brain (https://www.proteinatlas.org/ENSG00000178445-GLDC/brain). There are extensive historical and current data showing GLDC is indisputably expressed in astrocytes (as determined by immunohistochemistry of whole brain sections as well as molecular analyses of primary and cultured astrocytes (29, 30, 31, 32, 33). In addition, data from the brain protein atlas (https://www.proteinatlas.org/ENSG00000178445-GLDC/brain) and whole mouse brain RNAseq analyses (https://brainrnaseq.org/) suggest that GLDC is found in astrocytes, with very low levels in neurons and highly suppressed (or absent) in microglia. Notably, *Gldc*-knockout across all mouse brain neurons resulted in limited behavioral changes (of fear and anxiety) in the absence of hydrocephaly, neuromuscular disease, and fatality seen in NKH (34). In contrast germline-mutations or germline-knockouts in *Gldc* (expected to cause defective protein in both astrocytes and neurons) result in hydrocephaly, ventriculomegaly, and other neuromuscular hallmarks of prenataland postnatal fatal NKH disease (28, 35, 36). Our recent studies have established that attenuated mutant mice (*Gldc*^ATT/ATT^) show a 17 to 20% reduction in levels of astrocytes but no change in levels of neurons or microglia (35) suggesting a strong functional association between astrocytes and GLDC, in young attenuated mutant brains used in the current study. Thus, the attenuated *Gldc*^ATT/ATT^ mice are suitable to query whether astrocyte mitochondrial mechanisms of energy metabolism are regulated by GLDC and their persistence in severe mutants, all of which presently remain entirely undiscovered.

## Results

### Effects of *Gldc* mutation and animal age on brain glycine, serine, and responsiveness to folate deficiency

In prior studies, we developed a computational mutation scale to predict mutations linearly proportional to disease severity, based on which we engineered mice homozygous for A394V mutation (predicted to be attenuated; *Gldc*^*ATT*^) as well as a second mouse carrying G561R, S562F (close to the active site) expected to be severe (*Gldc*^*SEV*^) (28). Prediction of neurogenic severity was previously validated based on prenatal disease, but postnatal studies were not undertaken. Here, our goal was to compare metabolic and molecular differences of the brains of young and old pA394V attenuated (*Gldc*^ATT/ATT^) model of NKH as well as highly severe (*Gldc*^SEV/SEV^) model of NKH (28) to identify early mechanistic dysfunction seen in attenuated mutants and their persistence in severe mutants.

**Figure 1:**
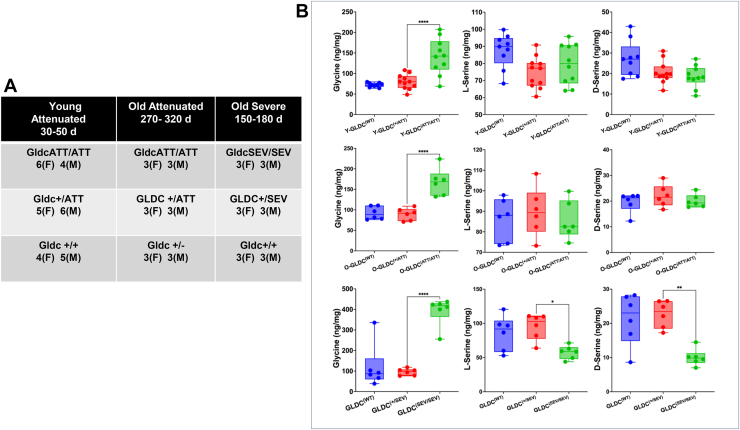
**Age and genotype specific alterations in glycine and serine levels in brain tissue.***A*, summary of experimental cohorts including young attenuated (4–6 week), old attenuated (9–11 months), and old severe (5–6 months) mice with indicated genotype and sex (M, males; F, females). *B*, LC-MS determination of L-serine and D-serine levels (in ng/mg) in brains of mutants (*green*) from young attenuated (Y-GLDC^ATT/ATT^), old attenuated (O-GLDC^ATT/ATT^) and severe (GLDC^sev/sev^) mutants, their wild type (*blue*) and heterozygous (*red*) counterparts. Median glycine levels of Gldc^(SEV/SEV)^ mice were 417.8 ng/mg), that of Y-GLDC^ATT/ATT^ and O-GLDC^ATT/ATT^ mice were 142.3 and 168.5 ng/mg, respectively, and wild type and heterozygous mice were at 90 ng/mg. Statistically significant reduction in levels of L- and D-serine in mutant mice compared to heterozygote counterparts were only seen for severe (GLDC^sev/sev^) mutants. Data are presented as individual values with medians ± SD. Significance was determined using one-way ANOVA. GLDC, glycine decarboxylase.

To understand the effects of both mutation severity and animal age, on absolute levels of postnatal brain glycine and serine, we compared brains from a total of 65 mice containing attenuated and severe mutants as well as age and gender-matched heterozygous and WT counterparts (Fig. 1*A*). For attenuated mutants we included two time points at ∼ 1 and ∼10 months of age, but for severe mutants we used a single time point of ∼5 months of age (due to difficulty in producing large numbers of severe mutants). As shown in Figure 1*B*, we found that median glycine levels in brains of severe mutants at 5 months increased to 417.8 ng/mg brain tissue, whereas attenuated mutants at 1 and 11 months presented medians of 142.3 and 168.5 ng/mg, respectively. In WT and heterozygous mice (across all ages), brain glycine levels ranged from 75 to 110 (with a median of 90 ng/mg). Together, these data suggested that in one month-old, attenuated mutants, brain glycine rose ∼1.5 fold. In severe mutants at 5 months, brain glycine was increased ∼3-fold compared to attenuated mutants at 1 month. Attenuated mutants at 11 months showed brain glycine increased by 2-fold relative to age-matched WT but were less than half relative to levels seen in severe mutants at 5 months. Together, these data suggested that although animal age may play a role, the severity of the *Gldc* mutation primarily determined the extent of glycine elevation.

Since serine and glycine can interconvert by the action of serine hydroxymethyl transferase 2 (SHMT2), we examined levels of L- and D-serine in mouse brains. Because heterozygotes are asymptomatic, we looked for significant differences between these mice and homozygous mutant counterparts. As shown in Figure 1*B*, compared to heterozygous mice, levels of neither L- nor D-serine were significantly reduced in attenuated mutants. However, L-serine was reduced in severe mutants (*p* < 0.05). Furthermore, the observed decrease in D-serine in severe mutants compared to heterozygotes was seen with a *p* value < 0.0012 (Fig. 1*B*). This suggested that severe mutants presented the greatest changes in L- and D-serine (compared to their corresponding heterozygote counterparts) and implicated that SHMT2 does not run the reverse conversion of glycine to serine in severe mutant brains.

**Figure 2:**
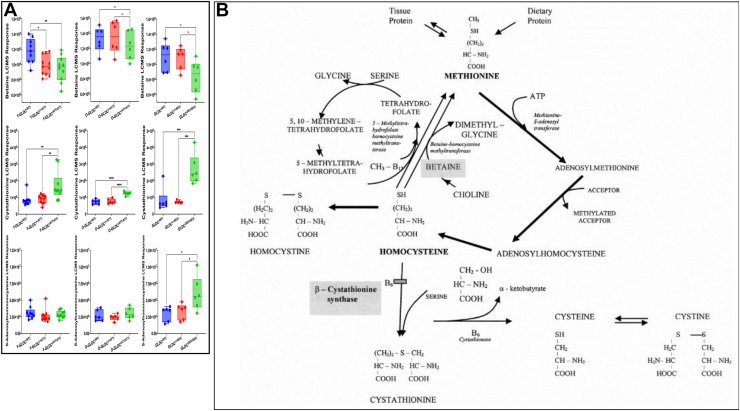
**Age and genotype specific changes in betaine, cystathionine, and S-adenosylhomocysteine (SAH) in the brain tissue.***A*, LC-MS response in young attenuated Y- Gldc^(ATT/AAT)^, old attenuated O- Gldc^(ATT/AAT)^, severe Gldc^(SEV/SEV)^ mutants (*green*), their heterozygote (*red*) and wild type (*blue*) counterparts. *B*, model summarizing severe and attenuated mutant responses. Severe mutants signal lack of 5-methyltetrahydrofolate and compensate by pushing elevated homocysteine toward cystathionine production, rather than continuing into the methionine cycle (which due to reduction of betaine is blocked in production of 5-meTHF). Compared to heterozygotes, attenuated mutants do not show a reduction in betaine or increase in SAH. Serine in attenuated mutants may drive limited increases in cystathionine.

We additionally found that severe mutant brains were reduced in betaine and increased in cystathionine and S-adenosylhomocysteine (SAH), while attenuated mutants only showed small increases in cystathionine (Fig. 2*A*). Based on the findings of Figures 1*B* and 2*A*, we concluded that, attenuated mutants (both young and old) show increases in glycine and likelihood of consuming serine in cystathionine production. However, the perturbations in these metabolites are low and do not result in marked change in pathways responsive to folate loss as revealed by no changes in levels of L- or D-serine, betaine and SAH. Thus notably, although our attenuated mutants brains show elevation of glycine, they do not show defect in folate pathways and associated metabolites. However, for severe mutants, there is a clear compensatory mechanism for the lack of 5-meTHF, and homocysteine is pushed toward cystathionine production rather than continuing the methionine cycle (which consumes a 5-meTHF unit), as proposed in the model in Figure 2*B*.

### Effects of *Gldc* mutation on the young attenuated NKH brain proteome

**Figure 3:**
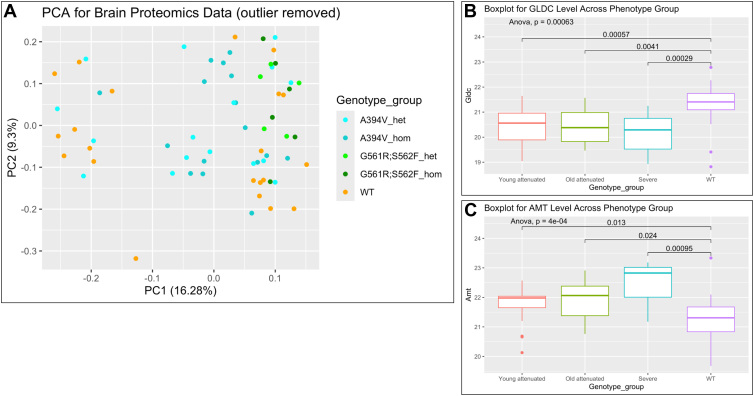
**Principal component and proteomic analyses of differential expression patterns in mouse brain across genotypes and phenotypes.***A*, PCA plots based on age and gender across ∼6000 brain proteins identified from wild type (WT), heterozygous (het) and homozygous (hom) mice expressing attenuated (A394V) or severe (G561R, S562F) mutations. *B*, boxplots of GLDC protein levels across indicated genotypes. *C*, boxplot of AMT protein levels across genotypes. *B–C*, boxplots represent interquartile ranges, with whiskers extending to the interquartile ranges and outliers shown as *dots*. Significance was determined using one-way ANOVA. AMT, aminomethyl transferase; GLDC, glycine decarboxylase; PCA, principal component analysis.

We next undertook proteomic analyses to gain broader understanding of mutation-induced molecular changes in the brain. We examined the whole brain proteome of all 65 mice. Details of preprocessing of the proteomic data and statistical analyses are provided in Experimental procedures and Table S1. Briefly, to identify proteins differentially expressed between disease and WT groups, we conducted age-matched two-sample *t* tests for each protein, stratified analyses by phenotype and age group. Proteins with nominal *p* values less than 0.01 were initially considered significant. However, none of these remained significant after multiplicity correction using the Benjamini–Hochberg method. We also performed principal component analysis (PCA) on proteomic results to evaluate separation of different groups. PCA assumes linear relationships among variables. In biological systems, especially in proteomics, interactions among proteins and pathways are often nonlinear. Subtle nonlinear shifts in expression patterns across groups may not necessarily be captured by PCA. In addition (and as shown in Fig. 3*A*), across ∼6000 brain proteins, we did not find clusters for age and gender. Combined with the limited sample size available for this study, we therefore opted to rely on unsupervised clustering methods (*e.g.*, hierarchical clustering). We next investigated changes in levels of GLDC and a second GCS protein, aminomethyl transferase (AMT), since variants in these two genes combined cause 99% of NKH. We found GLDC levels were reduced in brains of attenuated (young and old) as well as severe mutant mice (Fig. 3*B*). AMT was increased, particularly in severe mutants (Fig. 3*C*) consistent with much higher levels of compensatory responses for the lack of folates observed in these severe mutant mice (shown in Fig. 2).

**Figure 4:**
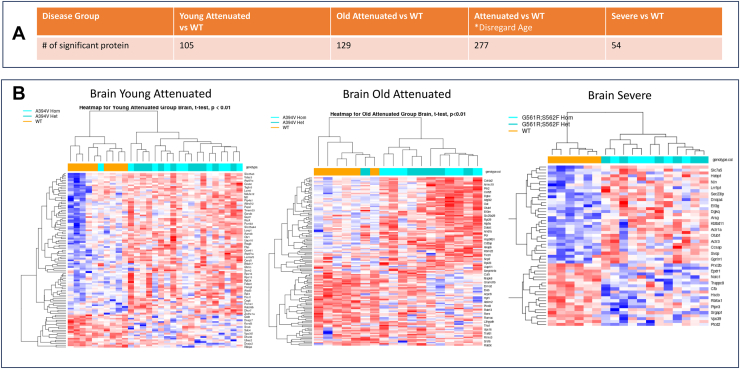
**Differential protein expression and unsupervised clustering based on phenotype.** Protein expression levels between age matched diseased group and WT group were investigated using *t* test. *A*, number of differentially expressed proteins in each phenotype-age group (*p* < 0.01). *B*, unsupervised clustering of the differentially expressed proteins for groups described in *A*. The homozygous mouse that clustered with wild type was confirmed by genotyping, suggesting young attenuated mutants show high degree of phenotypic heterogeneity. Graphs use mean imputed data for missing values; *t* test *p* < 0.01. Adjusted *p* values were not significant.

Differential protein analyses of the whole brain proteome based on phenotype were undertaken and are shown in Figure 4 (*p* < 0.01). The data suggested that brains of five-month-old severe mutants were better separated from their WT and heterozygote mice, relative to attenuated counterparts at 1 month or 11 months. Additional gene set enrichment analysis (GSEA) using threshold false discovery rate of 0.05 (Fig. S1) suggested young attenuated and old attenuated mutants showed enrichment in metabolic and stress pathways, whereas severe mutants additionally transitioned from metabolic compensation to glial and possibly neuronal pathology. However, due to limitations in generating sample sizes of severe mutants as well as older attenuated mutants, these predictions could not be experimentally verified in older attenuated or severe mutants. We were also unable to identify additional high value protein signatures shared across all three mutant phenotypes that separated them from heterozygotes and WT mice at *p* < 0.01, One reason for this may be that we used only six mutants from older attenuated and severe mutant groups, and these mice may present high levels of molecular heterogeneity intrinsic to differences in levels of *Gldc* deficiency. Notably, patients also show high levels of clinical heterogeneity with presentation of over 50 symptoms and no single symptom shared by all patients (20). Nevertheless, reduction of GLDC protein across all three proteomes, suggested this was the molecular basis for glycine elevation in brains of all attenuated and severe mutants (shown in Fig. 1). Sharp elevation seen in AMT in the severe mutant proteome (Fig. 3) supported that these mouse brains show prominent signatures of folate loss (as observed in Fig. 2).

### Effects of *Gldc* mutation on other GCS and PDH complex proteins

GLDC catalyzes the first and the rate-limiting step of glycine cleavage in the GCS. In this process it forms a complex with H protein (namely GCSH), which is needed for the transfer of the aminomethyl group released from glycine to the AMT protein. Dihydrolipoamide dehydrogenase, the fourth protein of the GCS, resets the glycine cleavage reaction by reoxidizing the lipoyl moiety of GCSH. Since GLDC has direct molecular interactions with GCSH (20), a reduction in the former may antagonize the stability of the latter. GCSH is a lipoyl transfer protein of the GCS, but it also mediates lipoylation of other mitochondrial proteins (37), including components of the PDH complex (38), which play key roles in mechanisms of energy metabolism in the brain.

**Figure 5:**
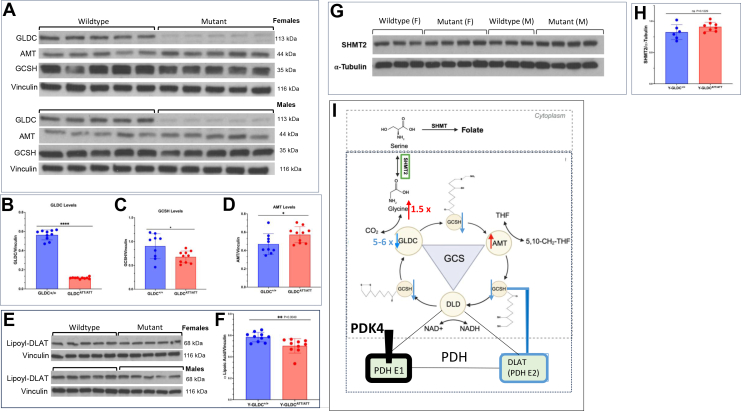
**pA394V mutation reduces levels of GLDC, GCSH, and lipoylation of DLAT.***A*, representative western blots showing GLDC, GCSH, and AMT in whole brain tissue from young attenuated mutants compared to wild type for female and male mice. *B–D*, corresponding quantification of GLDC (reduced 5-6-fold), GCSH (reduced 1.5-fold), and AMT in wild type and mutant brains. *E* and *F*, representative western blots showing lipoylated DLAT in brains of young attenuated mutants compared to wild type, for female and male mice and associated quantification. *G* and *H*, representative western blots showing SHMT2 in brains of young attenuated mutants compared to wild type, for female and male mice and associated quantification. *I*, a cellular model based on observed whole brain reduction in GLDC, GCSH, and lipoylation of DLAT (*blue*) increase in AMT (*red*) and no change in SHMT2. The model maybe relevant for astrocytes, where GLDC is expressed (along with GCSH and DLAT), but its relevance for neurons and microglia, where GLDC levels are low or unknown (https://www.proteinatlas.org/ENSG00000178445-GLDC/brain) (29, 30, 31, 32, 33) (https://brainrnaseq.org/) remains unclear. In astrocytes, PDK4 is expected to keep PDHE1α in a highly phosphorylated, inactivated state (*black*), and thus reduction in lipoylation of DLAT could trigger alternate mitochondrial mechanisms of energy metabolism. AMT, aminomethyl transferase; DLAT, dihydrolipoamide S-acetyl transferase; GLDC, glycine decarboxylase; PDK4, pyruvate dehydrogenase kinase 4; SHMT2, serine hydroxymethyl transferase 2.

To identify early mechanisms of mitochondrial energy mechanisms affected by mutations in GLDC, we focused on attenuated mutant mice aged 1 month. As shown in Figure 5, *A–D*, whole brains of these mice showed greater than 80% reduction of GLDC, strongly suggesting that GLDC was quantitatively reduced throughout the brain. We also found that whole brain levels of GCSH (a second protein of the GCS) decreased by 25%. In the first step of glycine cleavage, GLDC is known to form a covalent intermediate with GCSH, explaining why reduction in GLDC may suppress levels of GCSH. Because the GCS resides in mitochondria our finding of concomitant decrease of brain GLDC and GCSH implicated potential broader effects of NKH on energy mechanisms of mitochondria.

Since GCSH is a lipoyl-transfer protein, we considered whether the reduction in its levels may affect lipoylation of other mitochondrial proteins. We examined the effect on lipoylation of dihydrolipoamide S-acetyl transferase (DLAT), which forms the E2 subunit of the PDH complex (where dihydrolipoamide dehydrogenase provides the E3 subunit). As shown in Figure 5, *E* and *F*, lipoylation of DLAT was found to be measurably decreased (by 13%) in mutant compared to WT brains. In contrast, levels of AMT were increased by 15% and those of SHMT2 were unchanged (Fig. 5, G and *H*) consistent with our findings that young attenuated mouse brains did not show responsiveness to folates. Together these data strongly suggested that the observed depletion of GCSH and DLAT-lipoylation were due reduction in GLDC (and not AMT or SHMT2). We therefore summarize in Figure 5*I*, that depletion of GLDC (not changes in glycine and/or serine) antagonize levels of GCSH and lipoylation of DLAT, and both have potential to adversely impact energy mechanisms of mitochondria. Finally, although expression of GCSH and DLAT-lipoylation is expected to be distributed across all brain cells, a high proportion of GLDC (∼90%) is expected to reside in astrocytes. Moreover, we detect 80% reduction in GLDC in young attenuated mutant brains. Thus, we expect that astrocytes are likely to be most affected as described by our model in Figure 5*I* and this may impact mitochondrial energy mechanisms of astrocytes in the brain.

### Effects of *Gldc* mutation on astrocyte signatures of β−oxidation of fatty acids, glycolytic intermediates, and activation of PDH

**Figure 6:**
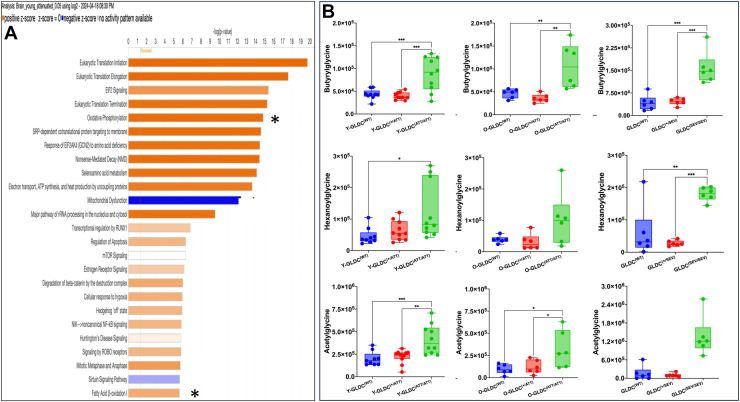
**Age and genotype specific changes in mitochondrial pathways of energy metabolism.***A*, ingenuity pathway analyses (IPA) predicts the proteome of young Gldc^(ATT/ATT)^ mice is enriched in two mitochondrial pathways of energy metabolism, namely oxidative phosphorylation and fatty acid β-oxidation activity. Pathways with significant enrichment are ranked by –log(*p* value), where *orange bars* indicate upregulated pathways, *blue bars* indicate downregulated pathways, and *gray* indicates no directional change. *B*, LC-MS response in changes in acylglycines (namely butyrylglycine and hexanoylglycine) as well as acetylglycine in young attenuated (Y- Gldc^(ATT/AAT)^), old attenuated (O-Gldc^(ATT/AAT)^) and severe (O-Gldc^(SEV/SEV)^) mutants (*green*), and their heterozygous (*red*) and wild type (*blue*) counterparts, suggesting mutants activate β-oxidation of short-chain fatty acids, an astrocyte mechanism of mitochondrial energy metabolism. GLDC, glycine decarboxylase.

Since DLAT is a key component of the PDH complex, reduction in its lipoylation is expected to activate mitochondrial mechanisms of energy metabolism. However, as shown in the model in Figure 5*I*, astrocytes do not activate their PDH, since this may antagonize neurometabolic support that astrocytes provide to neurons (39, 40). To identify alternate mitochondrial mechanisms, we undertook ingenuity pathway analyses of the attenuated mutant proteome (*p* < 0.05; Table S2). As shown in Figure 6*A*, we found that two of the top 20 altered pathways were mitochondrial pathways of oxidative phosphorylation and fatty acid β-oxidation. Oxidative phosphorylation is seen in both neurons and astrocytes, but the former prefer glucose as an energy source, whereas β-oxidation of short chain fatty acids is characteristic of activation of astrocyte mitochondria (16).

We therefore screened brains for signatures of production of short-chain fatty acid acids. As shown in Figure 6*B*, we followed hexanoylglycine, butyrylglycine, and acetylglycine as sensitive signatures of glycine capture by newly produced intermediates of β-oxidation of short-chain fatty acids and activated acetyl groups that could be easily differentiated between mutant and WT outputs. In young attenuated mutants, the levels of butyrylglycine and acetylglycine were significantly elevated compared to both WT and heterozygotes. Increase in hexanoylglycine was statistically significant only relative to WT. Analyses of old attenuated mutants and severe mutants showed additional increases in all three acylglycines and acetylglycine, with greatest changes seen in severe mutants (Fig. 6*B*). This suggested that fatty acid β-oxidation is stimulated as an early mitochondrial response in young attenuated mutants that become elevated as mice age or in response to increase in the severity of the mutation.

**Figure 7:**
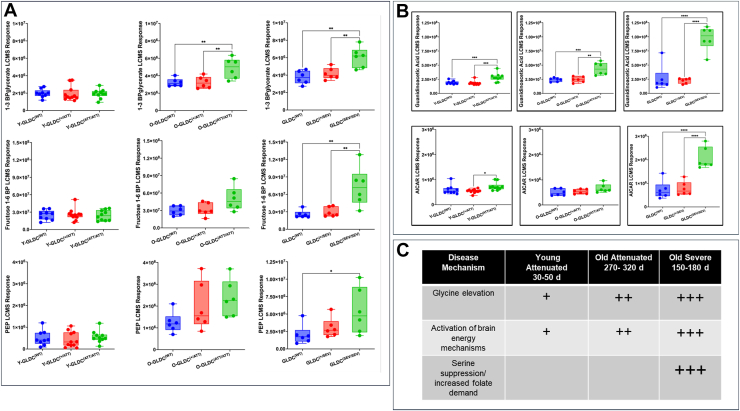
**Age and genotype specific changes in glycolytic intermediates and other factors of energy metabolism as well as a summary of distinct disease mechanisms in NKH attenuated and severe mice.***A*, LC-MS responses in changes in glycolytic intermediates in young attenuated (Y-Gldc^(ATT/AAT)^) old attenuated (O-Gldc^(ATT/AAT)^) and severe (O-Gldc^(SEV/SEV)^) mutants (*green*), and their heterozygous (*red*) and wild type (*blue*) counterparts. *B*, LC-MS responses in changes in GAA and AICAR in young attenuated (Y-Gldc^(ATT/AAT)^) old attenuated (O-Gldc^(ATT/AAT)^) and severe (O-Gldc^(SEV/SEV)^) mutants (*green*), and their heterozygous (*red*) and wild type (*blue*) counterparts. *C*, a summary of three different disease mechanisms seen in *Gldc*^*(ATT/AA*T^ and *Gldc*^*(SEV/SEV*)^mouse brains. Young attenuated mutants (expressing p.A394V which destabilizes GLDC) show two disease mechanisms, namely (i) mild increase in glycine and (ii) activation of brain energy mechanisms (reported in this study). Severe mutants (with double catalytic site mutations G561R,S562F) show three disease mechanisms, namely (i) highly (+++) elevated glycine (ii) high activation of brain energy mechanisms (reported in this study) and (iii) suppression of serine/increased folate dependency. AICAR, 5-aminoimidazole-4-carboxamide-1-β-D-ribofuranoside; GAA, guanidino acetic acid; GLDC, glycine decarboxylase; NKH, nonketotic hyperglycinemia.

We further examined glycolytic breakdown products of fructose-1,6-bisphosphate (F16P), 2,3-bisphosphoglycerate (2,3-BPG) and phosphoenolpyruvate (PEP) that occur in the cytoplasm as precursors to pyruvate (before it is imported into the mitochondria). As shown in Figure 7, *A* and *B*, we found glycolytic metabolites were sharply raised in severe mutants at 5 months. They increased to a lesser degree in old-attenuated mutants at 11 months. But notably, young attenuated mutants showed no increase in glycolytic intermediates reflecting utilization of glucose (and a signature of energy utilization by neurons) was not strongly perturbed. A stimulant of mitochondrial energy mechanisms, guanidino acetic acid (GAA), was significantly increased across attenuated and severe mutants (with highest levels associated with severe). A second stimulant, 5-aminoimidazole-4-carboxamide-1-β-D-ribofuranoside (AICAR) which signals starvation and acts as an activator of AMP-activate protein kinase (AMPK) was significantly raised in severe mutants compared to heterozygotes and WT mice, but not in attenuated mouse brains.

Together, the data in Figures 6 and 7, *A* and *B* suggested that an early, primary effect of *Gldc* mutation was stimulation of mitochondrial β-oxidation of short-chain fatty acids and activated acetyl groups in astrocytes (16) in absence of increase in glycolytic intermediates. Increased glycolytic intermediates were detected in older attenuated mutants and severe mutants and this is consistent with PDH dysfunction *via* decreased lipoylation. Elevation of GAA rather than AICAR suggests that GAA, a primary stimulant underlying the observed increase in signatures of mitochondrial oxidation of fatty acids in young attenuated mutants. That AICAR is not raised, suggests while the young attenuated mutant astrocytes activate alternate mechanisms of energy metabolism they are not yet starved (and neither are neurons). We summarized our findings as follows in Figure 7*C*. Notably, young, attenuated mutants (with the pA394V mutations) show two disease mechanisms of (i) mild elevation of glycine (well established by prior work in NKH) and (ii) activation of catabolic brain energy mechanisms (identified in this study). In contrast, severe mutants (with active site mutations) show three disease mechanisms, namely (i) highly elevated glycine (ii) increased activation of catabolic brain energy mechanisms of astrocytes as well as (iii) suppression of serine/increased folate dependency.

**Figure 8:**
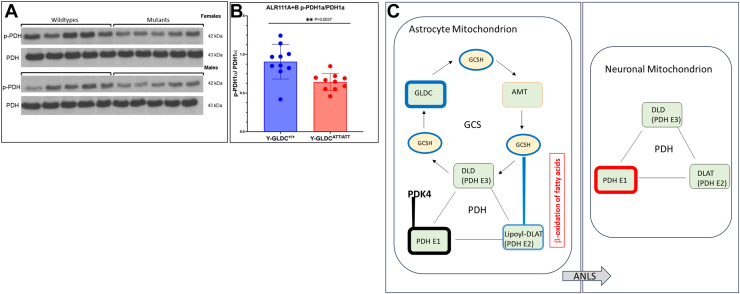
**Deficiency in GLDC decreases PDH phosphorylation.***A*, representative western blots showing levels of phospho-PDHE1α and total PDHE1α levels in whole brain tissue from young-attenuated mutants (Y-GLDC^ATT/ATT^) and wild type for female and male mice. *B*, corresponding quantification of the ratio of p-PDHE1α/PDHE1α in wild type and mutants. *C-D*, proposed model summarizing changes seen in the GCS and PDH complexes in the NKH brain. *C*, astrocytes. *blue* indicates reduction in GLDC, GCSH, and lipoylation of DLAT. AMT is raised (*red*) and SHMT2 is unchanged (not shown; Fig. 5). Astrocyte PDK4 keeps PDHE1α unchanged (*black*) in a highly phosphorylated state. -oxidation of short chain fatty acids is activated (*red*). *D*, neurons. Activation of PDH (*red*) detected in response to defect in astrocyte GLDC, suggests communication *via* the astrocyte-neuron lactate shuttle (ANLS). An alternative mechanism that could be proposed is that depletion of low levels of GLDC in neurons may affect glycolysis in brains of young attenuated mutants. However, the failure to detect changes in glycolytic intermediates, despite measuring elevation in astrocyte mitochondrial β-oxidation of fatty acids, favors the proposed ANLS mechanism shown above. AMT, aminomethyl transferase; DLAT, dihydrolipoamide S-acetyl transferase; GCS, glycine cleavage system; GLDC, glycine decarboxylase; NKH, nonketotic hyperglycinemia; PDH, pyruvate dehydrogenase; PDK4, pyruvate dehydrogenase kinase 4; SHMT2, serine hydroxymethyl transferase 2.

We wanted to further investigate the possible impact of catabolic mitochondrial energy mechanism on PDH in the brain. It is well established that in astrocytes, high levels of expression of pyruvate dehydrogenase kinase 4 (PDK4) phosphorylates the E1 subunit of PDH (to prevent pyruvate to acetyl-CoA conversion) even in the presence of activation of catabolic mechanisms of mitochondrial energy metabolism (18). Rather astrocytes convert pyruvate to lactate which can be transferred to neurons *via* the ANLS. This in turn increases pyruvate to acetyl-CoA conversion in neurons, driven by dephosphorylation of the neuronal PDH complex (41). As shown in Figure 8*A*, when we compared ratios of phosphorylated and unphosphorylated forms of PDH E1 forms in brains of mutant and WT mice, the mutants showed significantly lower levels of phosphorylation. Since PDK4 is expected to maintain high levels of PDH phosphorylation in astrocytes, the finding in Figure 8*A* may reflect activation of PDH in neurons. However, since neurons largely lack GLDC (https://brainrnaseq.org/), we propose that activation of neuronal PDH arises possibly through astrocyte-neuronal communication such as the ANLS shown in Figure 8*B*. Alternatively, it is possible that depletion of low levels of GLDC in neurons may affect glycolysis, but we fail to detect changes in glycolytic intermediates even as we see elevation in astrocyte mitochondrial β-oxidation of fatty acids in brains of young attenuated mutants (as respectively seen in Figures 7 and 6). Thus, in summary, the model in Figure 8*B* hypothesizes that quantitative reduction of GLDC, GCSH, and DLAT lipoylation in astrocytes coupled with PDK4-mediated heavy phosphorylation of PDHE1α limits pyruvate consumption in astrocytes *via* decarboxylation (18) but triggers alternate mechanisms of mitochondrial activation such as β-oxidation of fatty acids. In addition, the astrocyte’s increased conversion of pyruvate to lactate, is communicated *via* the ANLS to neurons where it drives activation of neuronal PDH.

## Discussion

Our study shows that mutations in *Gldc* that underlie NKH-type disease have deleterious consequences for brain energy metabolism, which has never previously been reported for either *Gldc* or NKH. We report that in attenuated and severe NKH mouse models, postnatal changes in brain glycine, D- and L-serine, betaine, cystathionine, GAA, and SAH are proportional to the severity of the mutation defect in GLDC. In young attenuated mouse brains, the direct enzymatic consequence was limited to 1.5-fold elevation in glycine. In these mice, there were no metabolite changes reflecting responsiveness to folate depletion or alterations in the levels of SHMT2. Yet the mouse brains showed greater than five-fold reduction in GLDC levels. Hence the relatively low (1.5-fold) increase in glycine may reflect the reverse action of SHMT2 that converts glycine to serine in attenuated mutants, which is transported out of the mitochondria and serves as a precursor of other amino acids, lipids, and nucleotides (as proposed in Fig. 5*I*). The massive reduction of GLDC protein levels may inflict nonenzymatic properties with deleterious consequences for brain energy metabolism. The wide spectrum of disease symptoms associated with NKH (20) is consistent for both enzymatic and nonenzymatic properties of mutant GLDC (and other GCS components) to play deleterious roles in the brain.

The nonenzymatic consequences of GLDC depletion evident in our mouse model were reflected by reduction in both GCSH and lipoylation of DLAT, a component of PDH. Defect in GCSH is known to cause NKH and lipoate deficiency (42). Decrease in lipoylation of DLAT with concomitant increase in signatures of β−oxidation of fatty acids confirmed that defect in GLDC activated alternate energy mechanism of astrocyte mitochondria. These changes seen in whole brain lysates suggest that they are widespread throughout the brain. They are seen in young attenuated, old attenuated and old severe mutants suggesting they occur early, from the onset of disease in the mouse and persist throughout postnatal development. These data and that GLDC is a major component of astrocytes which constitute 20 to 40% total cells in the brain (https://wwwncbinlmnihgov/books/NBK545142/) strongly support the models we propose in Figures 5*I* and 8*B* However, there are limitations of bulk tissue analysis undertaken in our studies, and thus future studies should assess whether astrocytes isolated from different brain regions at different animal ages show heterogeneity in levels of GLDC since this could impact their regional and age-dependent functions.

Prior studies have suggested that in lung cancer cells, pyruvate metabolism decreases on GLDC inhibition (43) In hepatocellular carcinomas elevation of GLDC increased lipoylation of DLAT to promote tumorigenesis (44) and knock down in GLDC decreased DLAT lipoylation to suppress PDH activation and tumor growth. We propose that activation of neuronal PDH occurs because of mutation of GLDC in astrocytes. A major function of astrocytes is to produce lactate and transport it to neurons, *via* the ANLS (45). This is achieved by blocking astrocyte PDH in a highly phosphorylated state which promotes pyruvate conversion to lactate (40, 46). Reduction of DLAT-lipoylation in mutant mice suggests how defects in GLDC may alter neuronal PDH activity (Fig. 8*B*). We note that our conclusions regarding astrocyte-specific *versus* neuronal-specific effects are based on prior knowledge of GLDC expression patterns and whole-brain lysates analyses. Furthermore, while the mechanism whereby astrocytic GLDC deficiency leads to neuronal PDH activation through the ANLS is persuasive, the model is currently supported by indirect evidence. Future cell type–specific isolation or single-cell studies may yield greater insights to dissect cell-specific biochemical changes. Since astrocytes are heterogenous population of cells that can be differentially activated, it will be important for future studies to assess whether difference in levels of GLDC also impact astrocyte inflammation and thereby mechanisms of disease.

Whether fatty acid oxidation-induced downstream stimulation of oxidative phosphorylation generates reactive oxygen species (16) that is detrimental to astrocytes also needs to be further investigated. Arribas-Carreira *et al.* 2024 (42) undertook differentiation of NKH patient (with human *GLDC* mutations)-induced pluripotent stem cells (iPSCs) into neural precursors and subsequently iPSC-derived astrocytes. They observed no difference in the oxygen consumption rate or cell viability, so the utility of testing for reactive oxygen species production in an *in vitro* system remains unknown. Nevertheless, these studies do suggest that *GLDC*-deficient neural precursor shift to more heterogenous astrocyte lineages and *in vitro* systems may be useful in looking at variation of GLDC levels across differentiated astrocytes.

Diseases caused by defects in mitochondrial enzymes frequently prevent mitochondria from producing enough energy. For instance, Leigh syndrome (27) (LS which affects approximately one in 40,000 individuals compared to NKH affecting one in 76,000) can arise from mutation in mitochondrially coded ATP6 (complex V), ND5 (complex I),CO2 (complex IV), or nuclear-coded enzymes such as SURF1 (complex IV), NDUFS4 (complex I), PDHA1 (pyruvate dehydrogenase complex) or COX10 (complex IV), and LRPPRC (complex III). Clinical presentation of Leigh syndrome is progressive neurological disease that includes seizures and hypotonia, symptoms that are shared with NKH, even though the core causes underlying the two conditions are different. Ketogenic diets that are shown to benefit severe and attenuated NKH patients (47, 48) have been ascribed to reduction of glycine, but they are also known to improve brain energy metabolism (49) which may contribute to their success in treating NKH and LS. By enabling neurons to use ketones, ketogenic diets may help reduce their dependence on the ANLS as well as tamp-back activation of β-oxidation of fatty acids caused by mutation in astrocyte GLDC. Our studies here provide the first evidence that GLDC directly plays a role in regulating mechanisms of mitochondrial energy metabolism in the brain and may provide new molecular targets to treat NKH disease.

## Experimental procedures

### Animals

CRISPR-Cas9 gene editing was used to introduce the attenuated (A394V, referred to as *Gldc*^*ATT/ATT*^) mutation in the C57BL6 strain (28). A restriction enzyme site (GTGAAC) for Hpy166II was created in the mutant allele. Tail biopsies were collected and genotyping was carried out by PCR-amplifying a 753 bp DNA fragment of *Gldc* flanking the mutation site using the following primers (forward: 5′-GTTGCATTTCCGTTTCTGGCT-3′ and reverse: 5′-ACTGCCCTCTTACTTGACCATT-3′), digesting the amplified product with Hpy166II and separating fragments by agarose gel electrophoresis.

Severe mutations (G561R, S562F) in the *Gldc* gene were introduced by CRISPR-Cas9 editing into C57BL/6 mice to create the *GldcSEV/SEV* strain (28). To create a restriction site for BsrG1, two silent nucleotide substitutions (TGCACC in WT changed to TGTACA in the mutant), were introduced downstream of the knock-in site. Genotyping was carried out as follows. A 728 bp DNA fragment of *Gldc* was PCR amplified using primers (forward: 5′-TGCTGTGCTGGGGAGAATTT-3′ and reverse: 5′-TGAACACAGCTACACTCAGCTT-3′), the amplified production was digested with BsrG1, and fragments were separated by gel electrophoresis. As previously reported (28) in the breeding of severe mutants, after 48 h of pairing, dams were separated, and sodium formate (30 mg/ml) was dissolved in sucralose (Portland) and provided in the drinking water: fomate-supplementation was discontinued once pups were born.

### Mouse brain proteome and metabolite analyses

Mouse brains were isolated and flash frozen and stored at −80 °C.

### Mouse brain proteome analyses

Cell pellets were resuspended in lysis buffer (8 M urea, 150 mM NaCl, and 50 mM Hepes, pH 8.5) and sonicated 3x for 30 s, with 30 s on ice between sonication cycles. Protein concentration was estimated by BCA (Pierce BCA Protein Assay Kit, Thermo Fisher Scientific). Hundred micrograms of denatured proteins were reduced with 5 mM DTT, alkylated with 15 mM iodoacetamide, and digested overnight with 1:100 LysC/Trypsin mixture (Thermo Fisher Scientific).

Peptides were analyzed using a Thermo Easy nLC 1200 coupled to a Thermo Orbitrap Eclipse mass spectrometer with data-independent acquisition. Peptides were separated using an EasySpray 50 cm column (Thermo Fisher Scientific). Solvent A was 0.1% formic acid in water, while solvent B was 0.1% formic acid in 80% acetonitrile. For each injection, we loaded 1 μg of peptides and separated them using a 90-min gradient from 2% to 28% B over 60 min, from 28% to 42% B over 15 min, from 42% to 100% B over 3 min, and held at 100% B for 12 min before re-equilibration. MS1 scans were collected at 60,000 resolution with a scan range of 390 to 1010 m/z, maximum injection time of 60 ms, and a normalized automatic gain control target of 400,000. MS2 scans were collected at 15,000 resolution in the Orbitrap mass analyzer. Precursor ions within a mass range of 400 to 1000 m/z were collected in 12 m/z isolation windows and fragmented using higher-energy collisional dissociation (HCD) collision energy of 30%. Mass spectrometry data were searched with FragPipe version 17.1 and the integrated DIA-NN module (50) against a spectral library generated from the UniProt mouse reference database (downloaded 2021–12–10). Methionine oxidation was set as a variable modification, and cysteine carbamidomethylation was set as a static modification.

### Quality control and normalization

Proteomic data were subjected to rigorous preprocessing steps to ensure data quality and reliability. Initially, features (proteins) with zero or missing values (NA) across all groups were excluded from the analysis. Subsequently, variance stabilizing normalization (vsn) (51) was applied to the dataset to correct for any systematic biases and to stabilize the variance across the measurements. Samples exhibiting low correlation within their respective groups were identified and removed from the dataset if their correlation coefficient fell below a threshold of 0.8. In this study, one sample was removed based on this criterion. In addition, features with low repeatability, characterized by a coefficient of variation greater than 0.3, were excluded to maintain data robustness. Missing values in the dataset were imputed using the mean value of all available samples to facilitate subsequent analyses. After quality control (QC) steps, the brain proteomics dataset consisted of 5975 features across 65 samples, reduced from an initial 6254 features before QC.

### Statistical analysis of differential protein expression and clustering

All statistical analyses were performed using R language in the RStudio environment. We conducted age-matched two-sample *t* tests for each protein analyses that were stratified by phenotype and age group. Initially, nominal *p* values less than 0.01 were considered significant, and the hits were subsequently subjected to multiplicity correction using the Benjamini–Hochberg method.

### Gene set enrichment analysis

GSEA was performed using the fgseaMultilevel function from the R fgsea package (52). For each comparison (young attenuated, old attenuated, and severe versus control), genes were ranked using a composite statistic: sign(log_2_ fold-change) × –log_10_(*p* value), prioritizing genes with both strong effect size and statistical significance. Reactome pathways were used as gene sets. Pathways were filtered to include only those with 15 to 500 genes to exclude very small sets prone to statistical noise and very large sets that may lack specificity. Enrichment scores were calculated using the standard scoring method (scoreType = "std"), and normalized (NES) to account for gene set size. Significance was assessed using permutation-based *p* values and adjusted for multiple testing using the Benjamini–Hochberg method. Pathways with false discovery rate < 0.05 were considered significant, and NES > 1.5 was interpreted as strong enrichment.

### Tissue metabolite analysis by LC-MS

Pulverized frozen tissue was spiked with 500 ng [^13^C_2_^15^N]-glycine and [^13^C_3_^15^N]-serine (Sigma-Aldrich) and extracted with ice cold 80/20 MeOH/water (40 μl/mg tissue). For absolute quantification of L/D-serine and glycine, a standard curve of each was run against the same 500 ng internal standard. Samples were spun at 10,000*g* for 10 min and supernatants transferred and dried prior to reconstitution in 50 μl water for LC-MS analysis (10 μl injection volume).

Positive and negative mode metabolite analyses were performed with reversed-phase ion-pairing liquid chromatography mass spectrometry (LC-MS) on a Thermo Vanquish Flex pump coupled to a QExactive orbitrap mass spectrometer using electrospray ionization (Thermo Fisher Scientific). Chromatography for negative mode ionization, the stationary phase was an ACQUITY UPLC HSS T3 (1.8 μm 2.1 × 150 mm) column. LC separation was achieved with a gradient elution of solvent A (97/3 H_2_O/methanol with 10 mM tributylamine, 15 mM acetic acid at a pH of 4.9), and solvent B (methanol). The gradient was 0 min, 0% B; 3 min, 20% B; 5.5 min, 20% B; 11 min, 55% B; 13.5 min, 95% B; 16.5 min, 95% B; and 17 min, 0% B. The flow rate was 200 μl/min. Positive mode LC separation was achieved with a gradient of solvent A (0.025% heptafluorobutyric acid, 0.1% formic acid in H_2_O) and solvent B (acetonitrile) at 400 μl/min. The stationary phase was a Waters Atlantis T3, 3 μm, 2.1 mm × 150 mm column. The gradient was 0 min, 0% B; 4 min, 30% B; 6 min, 35% B; 6.1 min, 100% B; 7 min, 100% B; and 7.1 min, 0% B. For both ionization modes, the injection volume was 10 μl, and the Q Exactive Mass Spectrometer scanned in negative mode from *m/z* 70-1000 at a resolving power of 70,000. For chiral analysis of serine, separations were performed with a Chirosil RCA (+) column (1.8 μm 4.6 x 150 mm). Separation was achieved with a gradient elution of solvent A (0.1% formic acid in water), and solvent B (0.1% formic acid in methanol). The gradient was 0 min, 0% B; 3 min, 20% B; 5.5 min, 95% B; 6 min, 95% B; 6.5 min, 0% B; and 8 min, 0% B. The flow rate was 600 μl/min.

### Western blots

Hemisected mouse brain tissue was lysed with tissue protein extraction reagent from Thermo Fisher Scientific and complete protease inhibitors (1 tablet/10 ml) from *Roche* (Fig. S2). Homogenized brain extracts were processed further *via* sonification using 5 s of pulsing and 5 s of rest for two rounds at 40 mA. The samples were then spun down at 14,000 rpms at 4 °C in 25 min. Protein concentration was quantified with a bicinchoninic acid assay (BCA, Thermo Fisher Scientific, 23225). Lysates were separated by SDS-PAGE at 150V for 65 min on a 10% acrylamide gel and transferred to a polyvinylidene fluoride membrane at 100V for 75 min. Membranes were blocked with 5% nonfat milk in 1X Tris-buffered saline with tween (TBST) for 1 h at room temperature. Primary antibodies in 1 × 5% nonfat milk were added to the membrane and probed overnight at 4 °C. A secondary antibody in 1 × 5% nonfat milk was added and kept on a rocker for 1 h at room temperature. Membranes were then washed (3x) off once again in 1X TBST and imaged with Pierce enhanced chemiluminescence reagents (700 μl, Thermo Fisher Scientific, 32106) and exposed using Hyperfilm ECL film (Cytiva, 28906839). Primary antibodies used were as follows: anti-GLDC (rabbit, Thermo Fisher Scientific, PA5-22101, 1:800), anti-Vinculin (mouse, Millipore, V9131, 1:4000), anti-AMT (rabbit, Thermo Fisher Scientific, PA5-76454, 1:3000), anti-GCSH (rabbit, Proteintech, 16726-1-AP, 1:1000), antiphosphorylated PDH (Ser232) (rabbit, Proteintech, 29582-1-AP, 1:6000), anti-PDH (rabbit, Proteintech, 18068-1-AP, 1:6000), anti-DLAT (mouse, Proteintech, 68303-1-Ig, 1:5000), anti-α Lipoic Acid (rabbit, Abcam, ab58724, 1:2000), and anti-SHMT2 (rabbit, Proteintech, 11099-1-AP, 1:800). Secondary antibodies were goat anti-mouse IgG (Bio-Rad, 1706516, 1:5000) and goat anti-rabbit IgG (Bio-Rad, 1706515, 1:5000).

### Statistical analysis

Statistical analysis was performed in GraphPad Prism (version 9.4.1) for both glycine analyses and western blots. The median levels ± SD were calculated for comparisons among treatment groups, and a two-way ANOVA with genotype and time as factors, followed by Tukey’s multiple comparisons test, was performed. A *p* value of <0.05 was considered significant (∗).

### Data availability

The mass spectrometry proteomics data have been deposited in the PRIDE ProteomeXchange Consortium through the MassIVE repository under the dataset identifier PXD075453 and MSV000101090. All of the remaining data described in the manuscript are located within the manuscript.

### Animal facility

We thank the Freimann Life Sciences Center (FLSC) and staff at the University of Notre Dame, for providing excellent services for mouse housing, caring, breeding, blood drawing, overall monitoring and veterinarian support.

## Regulatory approval

All procedures were approved by the Institutional Biosafety Committee (IBC), University of Notre Dame. Design of animal studies and procedures regarding their use were approved by the Institutional Animal Care and Use Committee (IACUC) University of Notre Dame.

## Supporting information

This article contains supporting information.

## Conflict of interest

The authors declare that they have no conflicts of interest with the contents of this article.
