# Supplementary material for: Variants in glycine decarboxylase activate catabolic mechanisms of mitochondrial energy metabolism in the brain
Source: J Biol Chem. 2026 Apr 27;302(6):113098. doi: 10.1016/j.jbc.2026.113098 (PMC13235479; doi:10.1016/j.jbc.2026.113098)
Supplement: Legends for Tables S1 and S2 [file mmc1.docx]

**Legends for Supplementary Tables S1 and S2.**

**Supplementary Table S1. Master brain proteome.** Proteome data from brain tissues sample were undertaken as previously described^35^. and summarized in Materials Methods and Results. Briefly, proteins with zero or missing values (NA) were excluded and variance stabilizing normalization (vsn)^36^ was applied to correct for any systematic biases and after removal of samples whose correlation coefficient and repeatability were low (respectively < 0.8. and coefficient of variation > 0.3) The brain proteomic dataset shown consisted of 5975 features across 65 samples, reduced from an initial 6254 features before QC.

**Supplementary Table S2. Brain proteome for IPA analyses.** Proteome of brains of young-attenuated mice relative to heterozygous and wild type countered, filtered p < 0.05 were used in Ingenuity Pathway Analyses (IPA), data for which is shown in Fig. 6A.
