# Supplementary material for: Variants in glycine decarboxylase activate catabolic mechanisms of mitochondrial energy metabolism in the brain
Source: J Biol Chem. 2026 Apr 27;302(6):113098. doi: 10.1016/j.jbc.2026.113098 (PMC13235479; doi:10.1016/j.jbc.2026.113098)
Supplement: Figure S1 [file mmc2.docx]

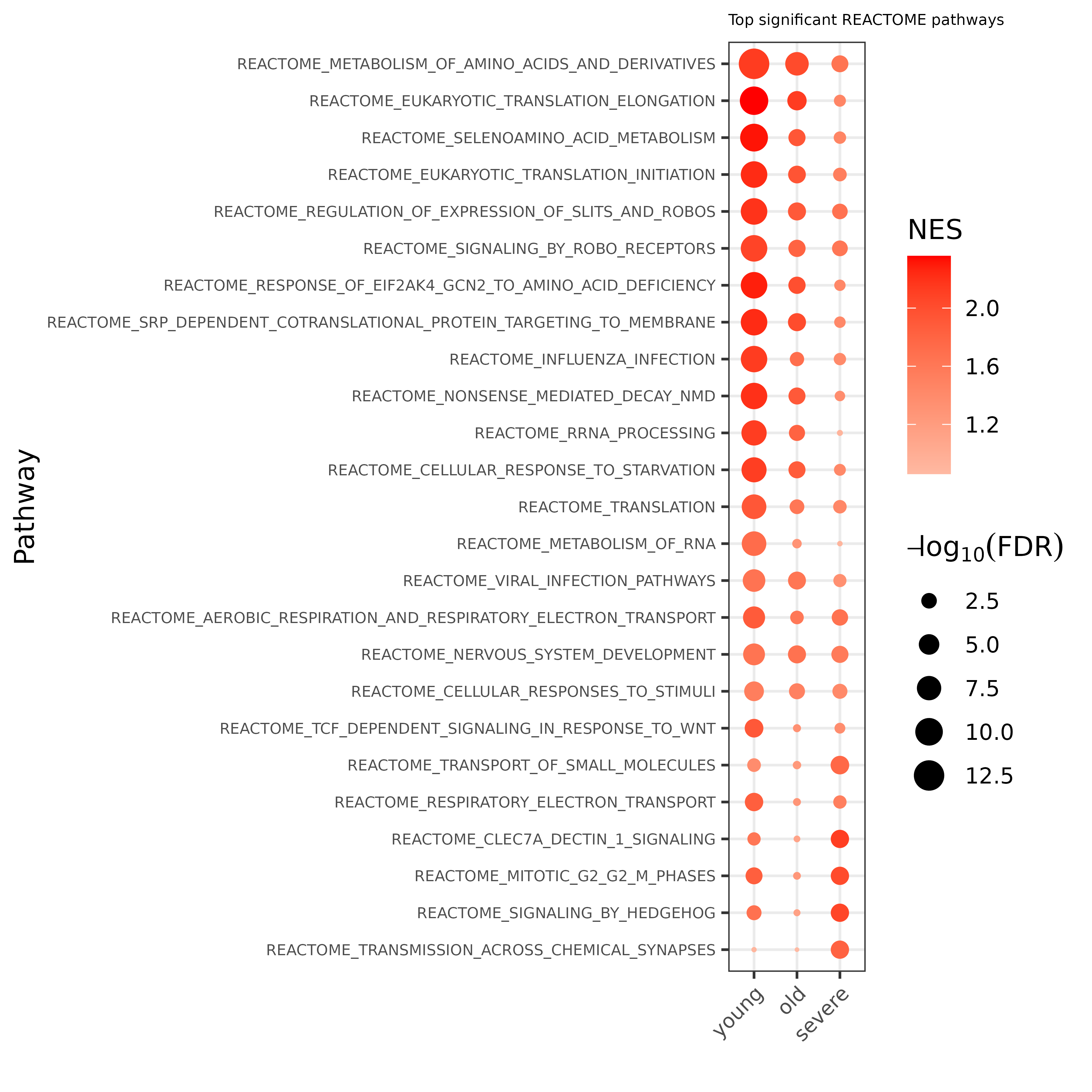


**Supplementary Figure S1. Gene Set Enrichment Analysis (GSEA).** Coordinated biological responses to GLDC deficiency in non-ketotic hyperglycinemia (NKH), were investigated via performed preranked GSEA) using Reactome pathways across three comparisons: young attenuated, old attenuated, and severe GLDC mutant mice versus controls. Applying a false discovery rate (FDR) threshold of 0.05 yielded 24 significant pathways in young, and 17 each in old and severe comparisons. The most significantly enriched pathway across all groups was Metabolism of amino acids and derivatives (NES: 2.13 in young, 2.02 in old, 1.65 in severe; FDR < 0.001). Additional enrichment of Selenoamino acid metabolism and Response of EIF2AK4/GCN2 to amino acid starvation (NES > 2.2 in young and old NES ~2) indicates activation of amino acid sensing and one-carbon metabolism stress responses. Young and old mutants also showed strong enrichment of Eukaryotic translation elongation, translation initiation, SRP-dependent cotranslational targeting, and nonsense-mediated decay (NES up to 2.2; FDR < 0.001). These were accompanied by significant enrichment of Cellular response to starvation, consistent with activation of the integrated stress response (ISR). Pathways such as Nervous system development, Slit/Robo signaling, were enriched only In severe mutants, as were additional enrichment of Transmission across chemical synapses, Transport of small molecules, and CLEC7A/Dectin-1 signaling was observed, indicating involvement of synaptic, developmental pathways. In our dataset, all NES values were positive indicating that enriched pathways were upregulated in mutants. We considered pathways with FDR < 0.05 as statistically significant, and those with FDR < 0.1 as suggestive trends. NES > 1.5 was interpreted as a strong effect. Each row in the results file corresponds to one Reactome pathway and includes the following. pathway: pathway name; NES: normalized enrichment score; pval: nominal p-value; padj: FDR-adjusted p-value; size: number of genes in the set; condition: comparison group (young, old, severe); NegLog10Padj: –log10(padj), used for visualization
