# Supplementary figures and images for: Variants in glycine decarboxylase activate catabolic mechanisms of mitochondrial energy metabolism in the brain
Source: J Biol Chem. 2026 Apr 27;302(6):113098. doi: 10.1016/j.jbc.2026.113098 (PMC13235479; doi:10.1016/j.jbc.2026.113098)

Figure 5A raw films

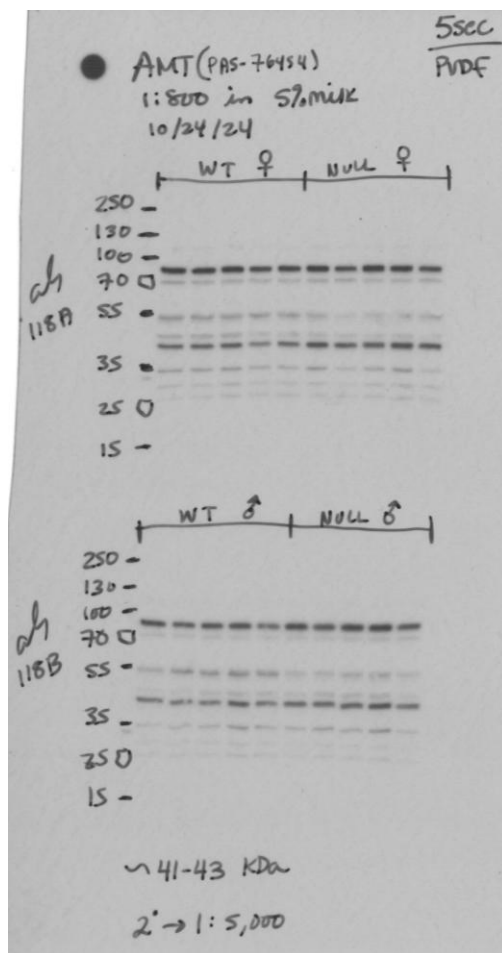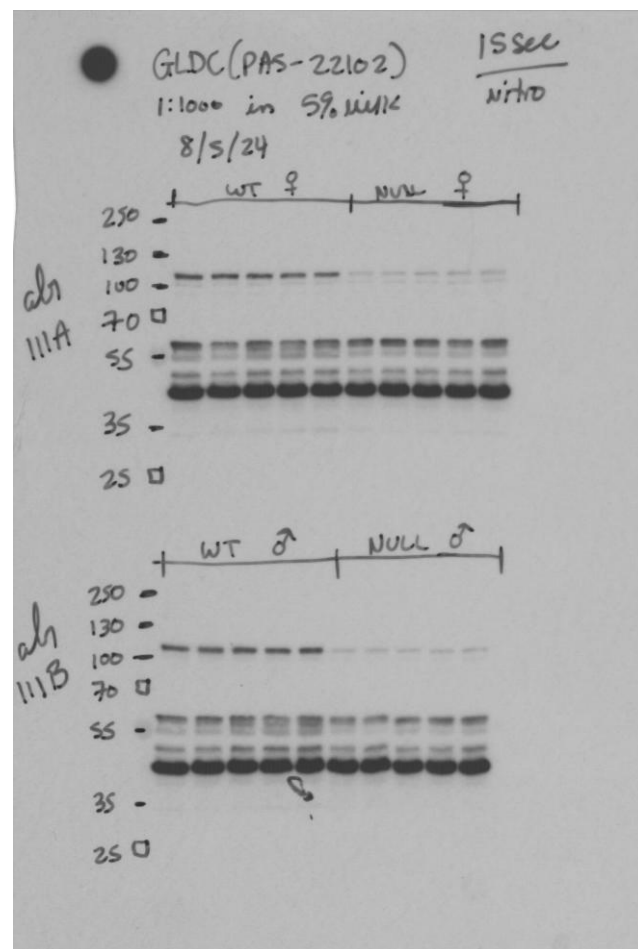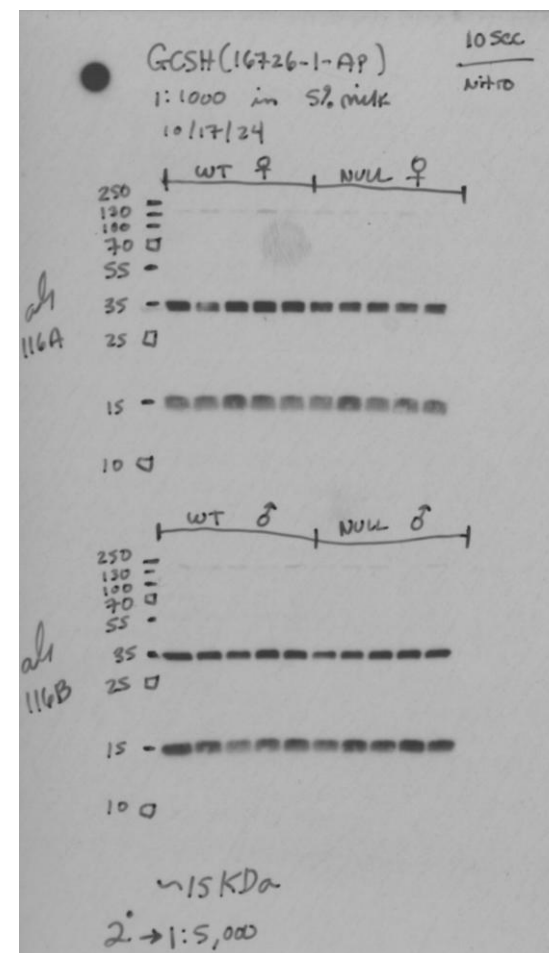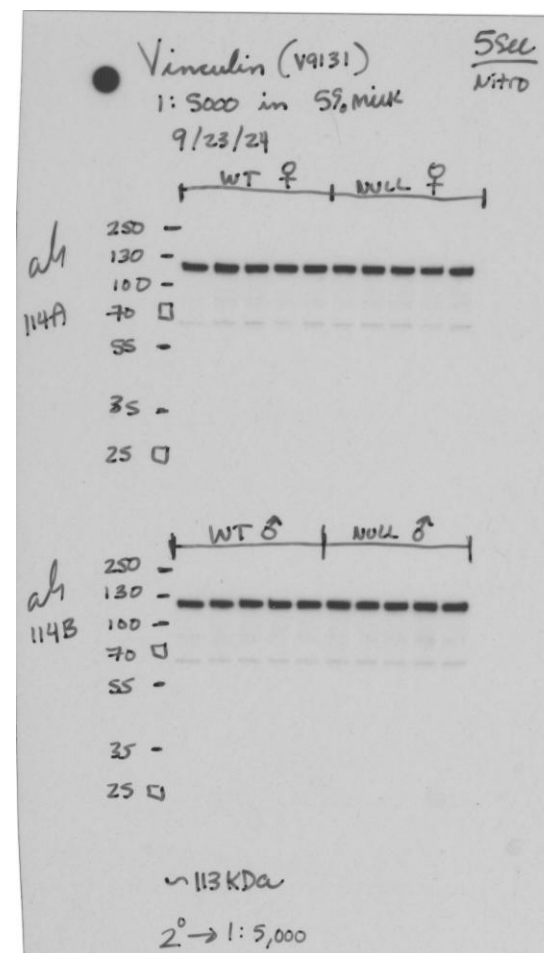

Figure 5E raw films

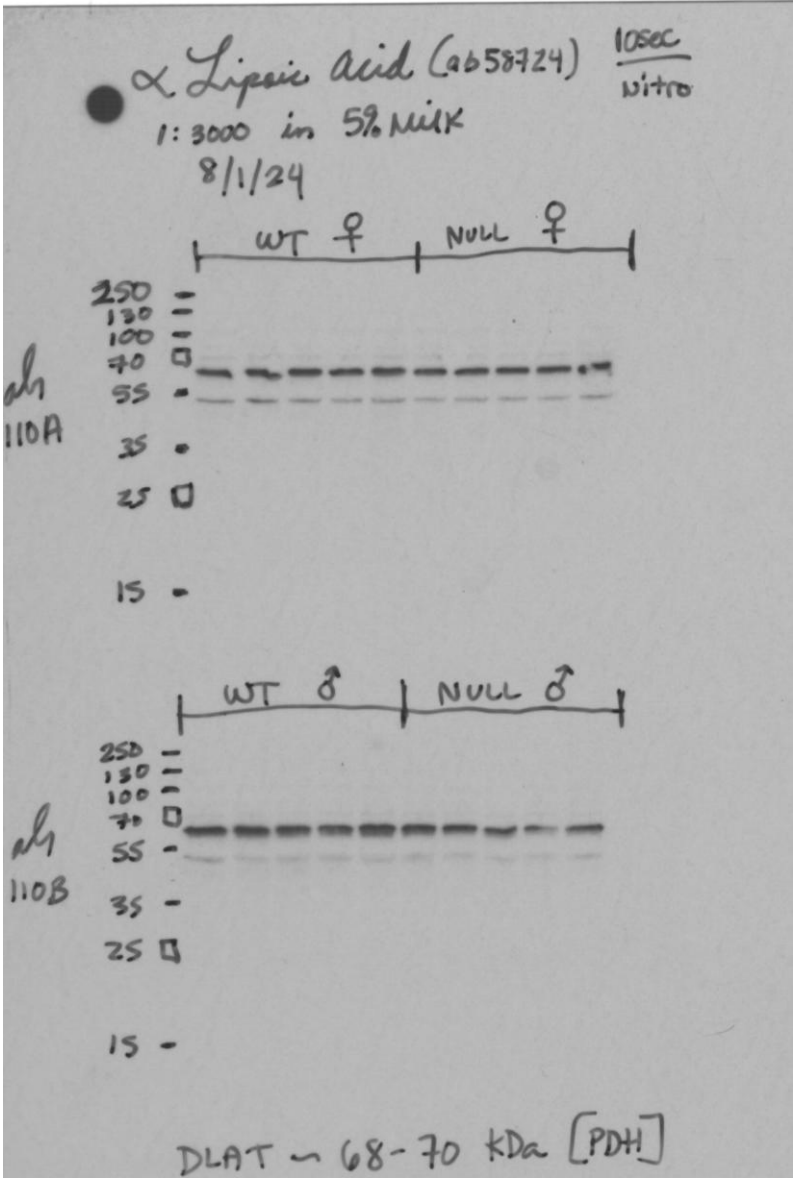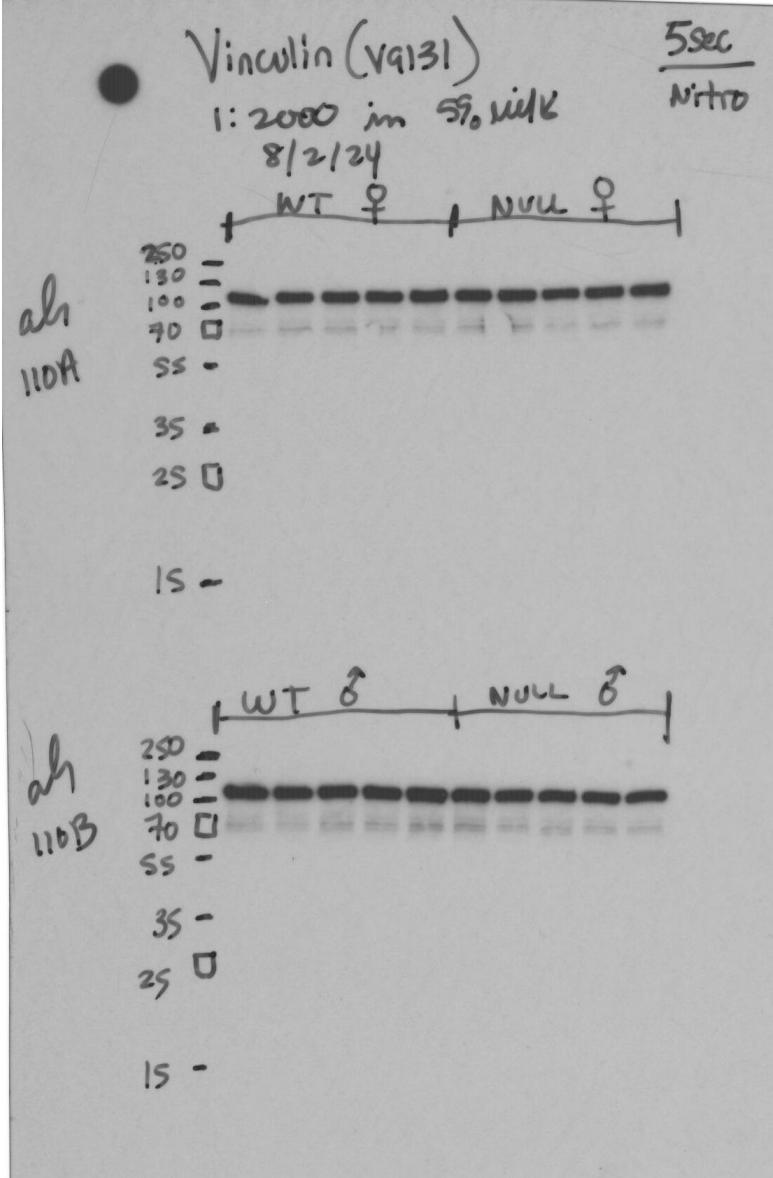

Figure 5G raw films

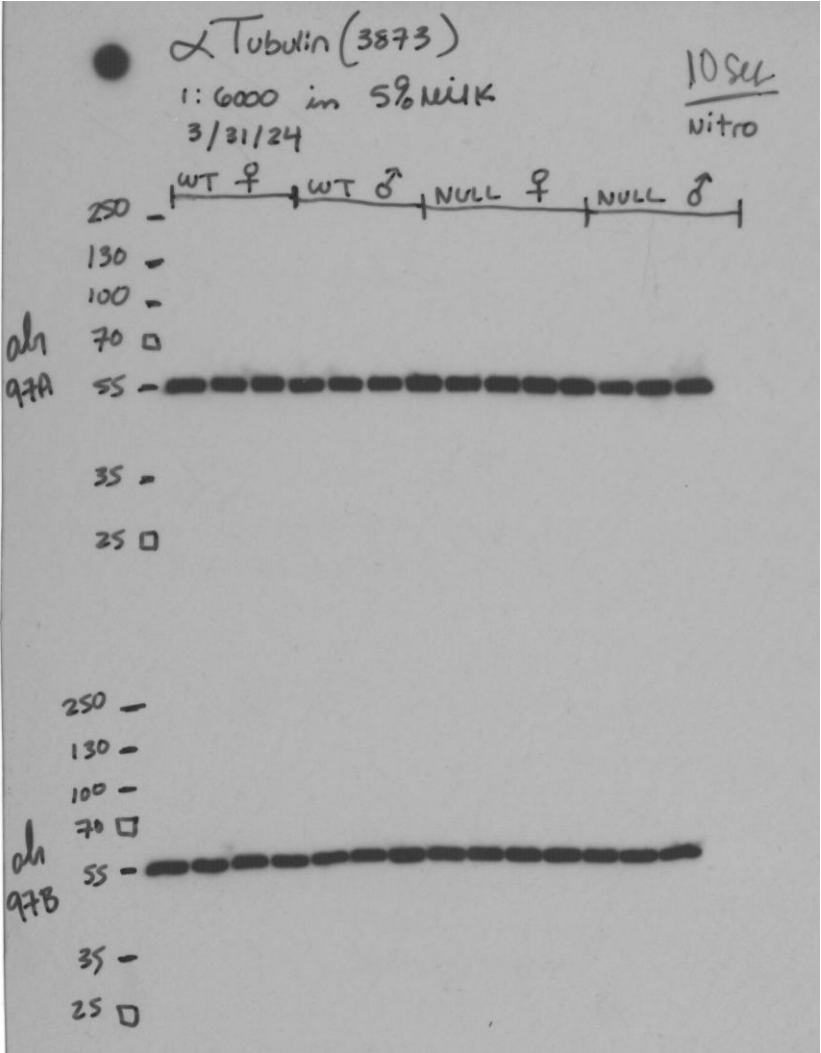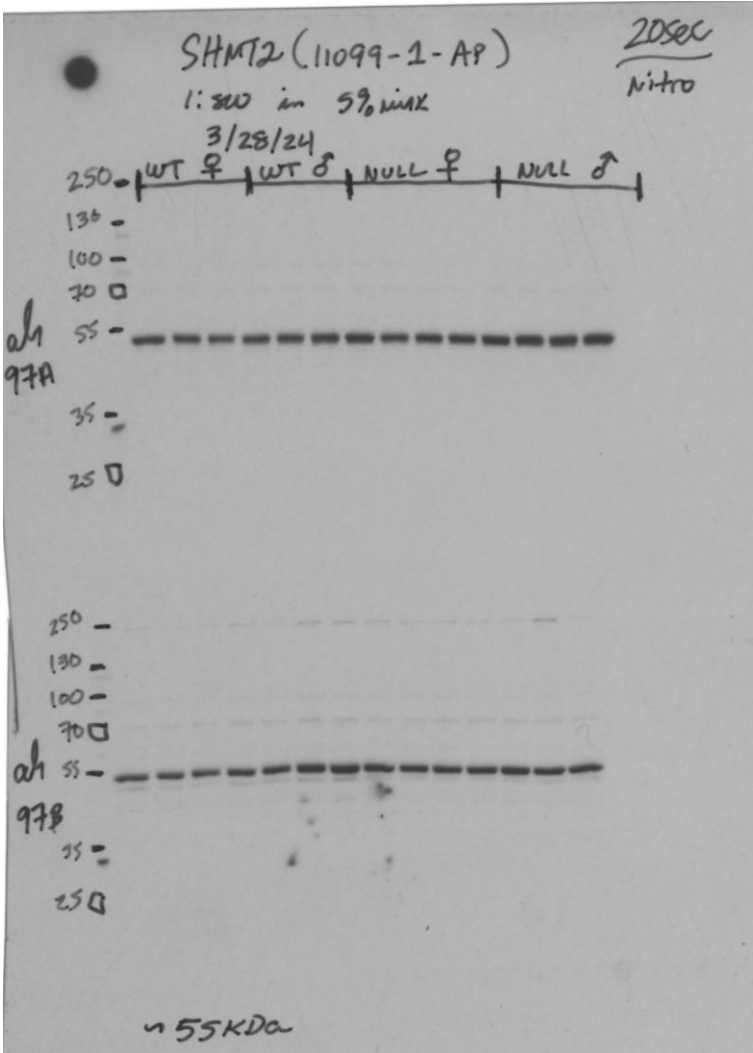

Figure 8A raw films

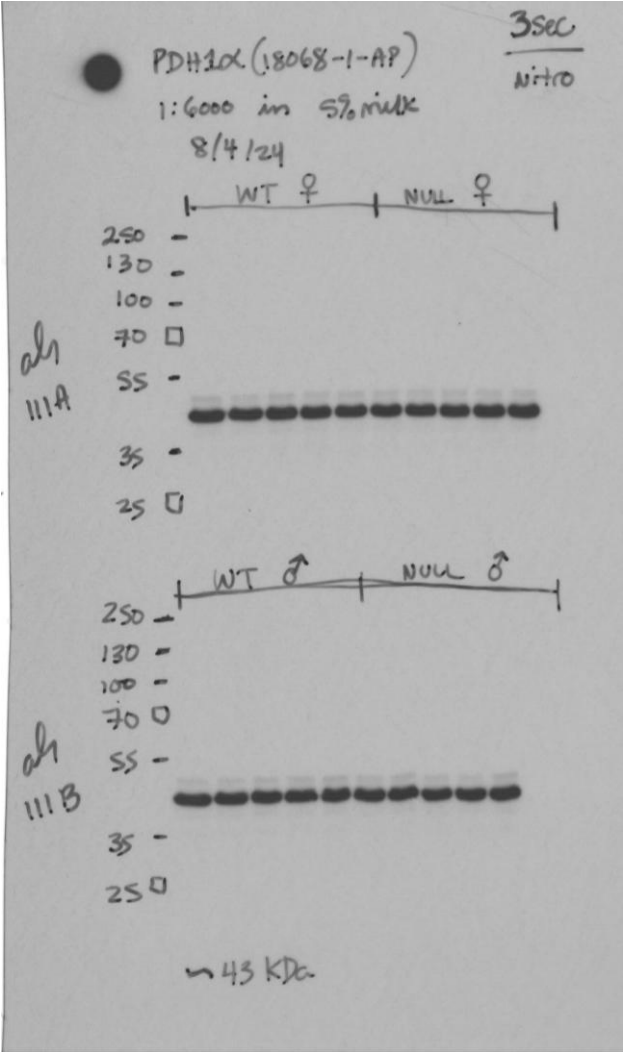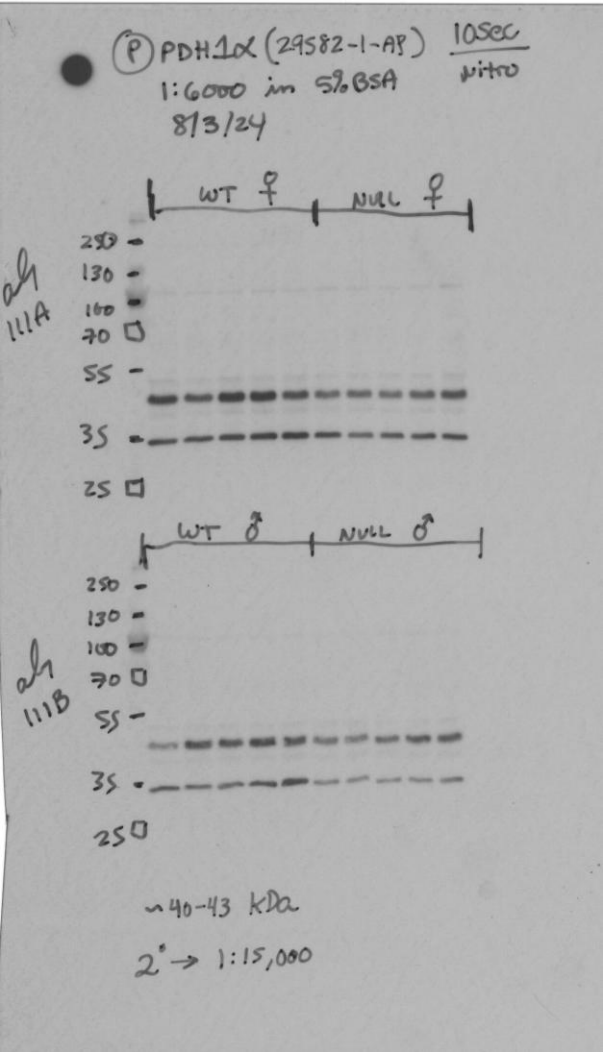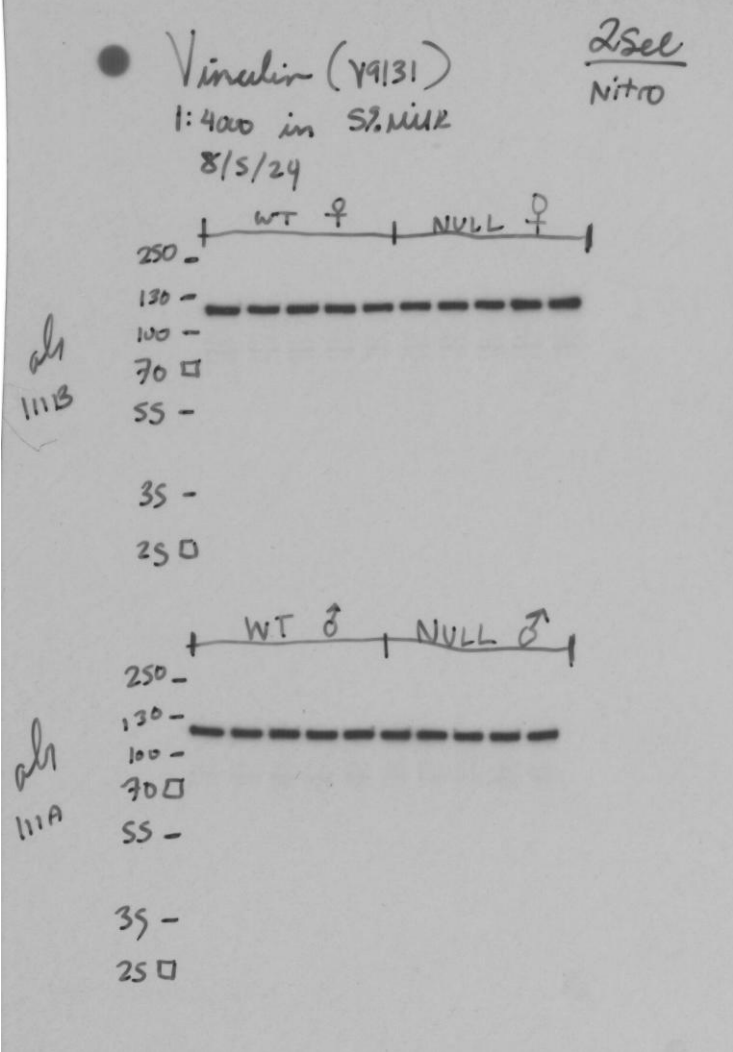

Supplement: Figure S2 [file mmc3.pdf]
